# Comparison of Robotic Versus Conventional Open Thyroidectomy for Recurrent Laryngeal Nerve Safety: A Systematic Review and Meta-Analysis

**DOI:** 10.7759/cureus.53860

**Published:** 2024-02-08

**Authors:** Christos Sialakis, Aikaterini Frantzana, Christos Iliadis, Petros Ouzounakis, Panagiota Antoniou Sialaki, Lambrini Kourkouta

**Affiliations:** 1 Otolaryngology, Private Diagnostic Health Center, Thessaloniki, GRC; 2 Epidemiology and Public Health, George Papanikolaou General Hospital of Thessaloniki, Thessaloniki, GRC; 3 Public Health, European University Cyprus, Nicosia, CYP; 4 Nuclear Medicine, Private Diagnostic Health Center, Thessaloniki, GRC; 5 Medicine, General Hospital of Alexandroupolis, Alexandroupoli, GRC; 6 Internal Medicine, Limassol Hospital, Limassol, CYP; 7 Nursing, International Hellenic University, Thessaloniki, GRC

**Keywords:** recurrent laryngeal nerve, robotic thyroidectomy, conventional thyroidectomy, open thyroidectomy, thyroidectomy

## Abstract

This review aims to investigate the safety of recurrent laryngeal nerve (RLN) by comparing robotic thyroidectomy (RT) versus open thyroidectomy (OT) in Western and Asian populations. Two main outcomes of this review were (1) the safety of RLN comparing the robotic and OT assessing transient and permanent laryngeal nerve (PLN) palsy as a postoperative complication in each surgical procedure and (2) the safety of RLN comparing the robotic and OT assessing transient and permanent laryngeal nerve (PLN) palsy as a postoperative complication between studies conducted in USA/Europe and Asia. We searched relevant literature in electronic databases such as PubMed, MEDLINE, Cochrane CENTRAL, ScienceDirect, and Cumulative Index to Nursing & Allied Health (CINAHL) up to September 2022. Further research was performed during January 2024 in the Scopus database. Two primary outcomes were set: transient RLN palsy and permanent RLN palsy, comparing RT and OT.

In this review, 18 non-randomized studies were included. A statistically significant difference between robotic and conventional OT was not observed either in transient RLN or in permanent RLN palsy. The odds ratio (OR) for the overall comparison of transient RLN palsy was 1.18, and the 95% confidence interval (95% CI) was 0.80-1.74. The subgroup analysis for transient RLN palsy between USA/Europe studies was OR 1.28, and the 95% CI was 0.64-2.58. The subgroup analysis for transient RLN palsy between Asian studies was OR 1.14, and the 95% CI was 0.72-1.82. The OR for the overall comparison of permanent RLN palsy was OR 0.90, and the 95% CI was 0.38-2.15.

The subgroup analysis for permanent RLN palsy between USA/Europe studies was OR 0.45, and the 95% CI was 0.07-2.97. The subgroup analysis for permanent RLN palsy between Asian studies was OR 1.13, and the 95% CI was 0.42-3.05. Heterogeneity I^2 ^was 0% in all outcomes. The Mantel-Haenszel method fixed effect was used. First, RT and open conventional thyroidectomy have comparable safety for RLN, although the analysis showed no statistically significant results. Second, no statistically significant results were found for RLN safety in either USA/Europe or Asian studies. Considering that there is not a statistically significant difference between the two approaches for RLN safety, and due to the limited number of studies from Western countries, the results should be considered with caution. Important factors such as the patient’s body characteristics, the existing thyroid pathology, and the surgical approach should be kept in mind. More comparable studies are needed on the Western population.

## Introduction and background

In the last few decades, an increasing improvement in the surgical techniques for thyroidectomy has been observed. Robotic thyroidectomy (RT) has become popular around the world for surgeons and patients who are interested in a procedure that allows the removal of the thyroid gland with a better cosmetic result when compared to the conventional open thyroidectomy (OT) approach [1]. The safety of recurrent laryngeal nerve (RLN) got special attention, and for this reason, it is necessary to identify nerve anastomoses and the external branch of the superior laryngeal nerve [2].

Thyroidectomy has a long history, beginning from 1873 to 1893 when Billroth and Kocher standardized the operative procedure for thyroidectomy. The technique was adopted by head and neck surgeons [3]. Some of the complications related to total thyroidectomy are permanent hypoparathyroidism and RLN injury. The incidence of permanent recurrent laryngeal nerve injury has varied from 0% to 14% and from 1.2% to 11% for permanent postoperative hypoparathyroidism [4]. An important factor associated with successful RT is the experience and skill of the surgeon. There are many approaches to RT. Strict selection criteria are necessary for a successful operation and to minimize complications [1].

RT overcomes the two-dimensional limitations associated with endoscopic thyroidectomy. Furthermore, RT enables the usage of a three-dimensional camera and articulated instruments, reducing the physiological tremor of the surgeon. As a result, RT reduces the limitations of traditional OT [3]. RT consists of three steps. The first stage is the formation of the working space and patient positioning; the second is the docking stage, which includes the positioning of the robotic arms; and the third is the console stage [1,3].

Indications for RT and neck dissection are as follows: follicular tumors with a size of 5 cm or less and differentiated thyroid cancer. Patients are excluded if they have thyrotoxicosis, other pathologic conditions of the neck and shoulder, a history of previous head and neck surgery or irradiation, the possibility of perinodal infiltration to an internal jugular vein or major nerves, and distant metastasis [3]. RT continues to develop. Careful selection of patients reduces complications and enhances success. Nevertheless, it is important to note that the operation should be performed by an experienced robotic surgeon [1].

## Review

Methods

This study adheres to the guidelines of the Preferred Reporting Items for Systematic Reviews and Meta-Analysis (PRISMA) 2020 checklist [5].

Inclusion and Exclusion Criteria

Inclusion criteria were set as follows: (1) retrospective and prospective studies, (2) patients operated with an open conventional thyroidectomy compared to an RT, and (3) studies conducted in the English language. Exclusion criteria were set as follows: (1) studies that are not cohorts, (2) non-English studies, and (3) studies that include patients who have undergone other surgical operations different from open or RT.

Information Resources and Search Strategy

An electronic search was performed on PubMed, ScienceDirect, Cumulative Index to Nursing & Allied Health (CINAHL), MEDLINE, and Cochrane CENTRAL. The last search was performed on September 2022, using keywords such as “thyroidectomy,” “open thyroidectomy,” “conventional thyroidectomy,” “robotic thyroidectomy,” and “recurrent laryngeal nerve.” Further research was performed on Scopus in January 2024 using the keywords such as “thyroidectomy,” “open thyroidectomy,” “robotic thyroidectomy,” “western population,” “Europe,” “America,” and “recurrent laryngeal nerve.”

Selection Process and Data Collection Process

Two authors worked independently on abstract and full-text screening. From the included studies, collected data were about the type of thyroidectomy performed, year of publication, and sample size of included studies. For the data collection process, the authors worked independently. Any disagreements during the selection and data collection process were resolved by discussion.

Effect Measures

In this review, we compared the safety of robotic versus OT. We measured the following outcomes: (1) transient laryngeal nerve injury and (2) permanent laryngeal nerve injury. The effect measures were calculated using the odds ratio (OR), Mantel-Haenszel (M-H) analysis [6], and 95% confidence intervals (95% CIs), with fixed effect.

Evaluation of the Quality of Included Studies

Two authors independently used the Methodological Index for Non-randomized Studies (MINORS) [7] to assess the quality of the included studies. The main points of evaluation were the following: (1) a clearly stated aim, (2) inclusion of consecutive patients, (3) prospective collection of data, (4) endpoints appropriate to the aim of the study, (5) unbiased assessment of the study endpoint, (6) follow-up period appropriate to the aim of the study, (7) loss to follow-up less than 5%, (8) prospective calculation of the study size, (9) an adequate control group having a gold standard diagnostic test or therapeutic intervention, (10) contemporary groups, (11) baseline equivalence of groups, and (12) adequate statistical analyses.

The items are scored 0 (not reported), 1 (reported but inadequate), or 2 (reported and adequate).

Statistical Methods

Statistical analysis includes the calculation of the OR for the dichotomous variables using the Mantel-Haenszel [6] method with fixed effect, 95% CIs, test for overall effect Z, and heterogeneity I^2^ [8]. All parameters were calculated using the RevMan 5.4.1 software package [9]. For heterogeneity, we considered 0%-40% as a low degree, 41%-75% as a moderate degree, and 76%-100% as a significant degree.

Results

**Figure 1:**
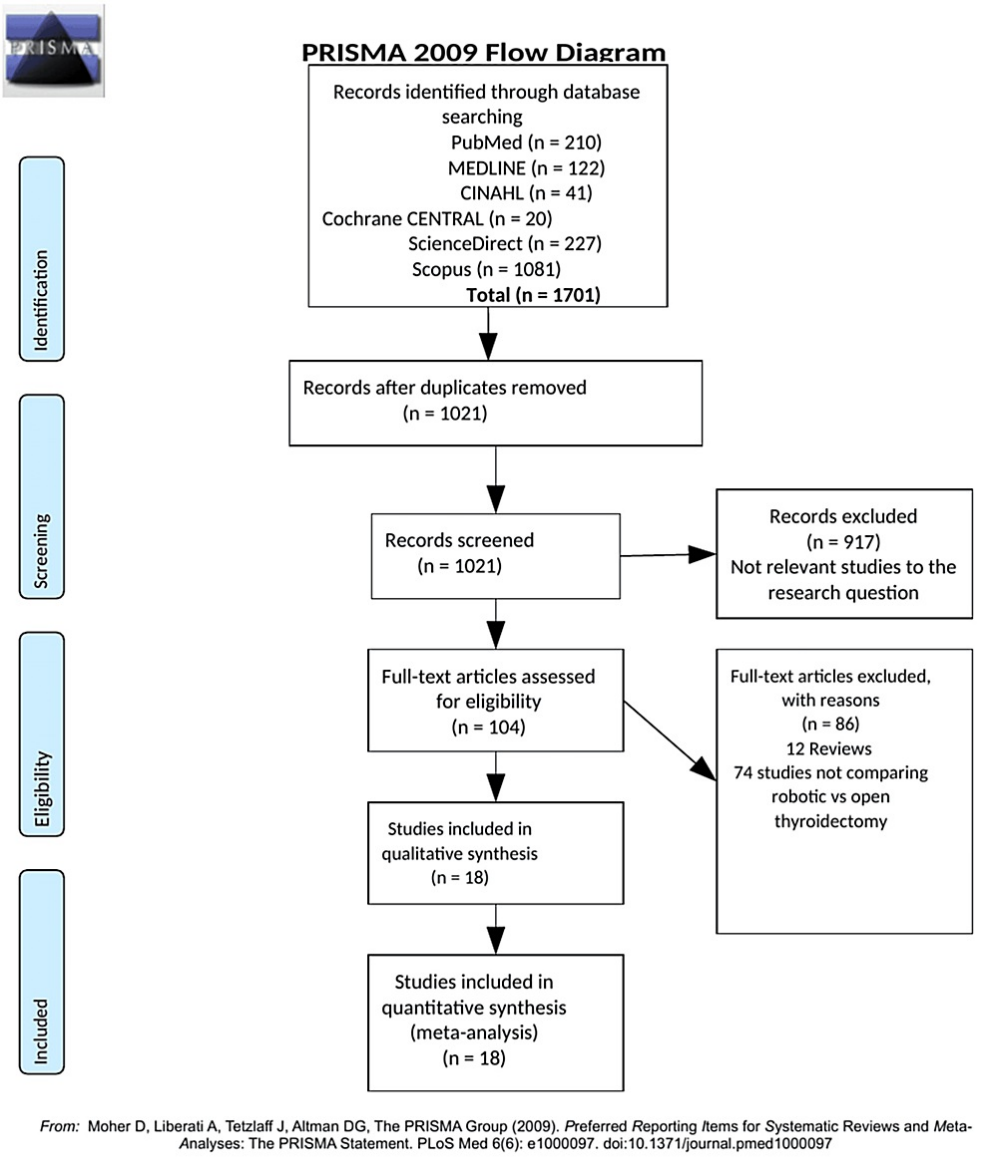
PRISMA flow diagram Source: Ref. [10]. PRISMA: Preferred Reporting Items for Systematic Reviews and Meta-Analysis; CINAHL: Cumulative Index to Nursing & Allied Health.

A flow diagram that shows the screening process is shown in Figure 1 [10].

Table 1 shows the characteristics of the included studies.

**Table 1:** Characteristics of the included studies Source: Refs. [7,11-28]. BABA: Bilateral axillo-breast approach; TAA: Transaxillary approach; BAA: Bilateral axillary approach; GUA: Gasless unilateral axillary approach; GUAB: Gasless unilateral axillo-breast approach; RT: Robotic thyroidectomy; OT: Open thyroidectomy; BMI: Body mass index; MINORS: Methodological Index for Non-randomized Studies.

Study	Study type/Country	Number of patients	Robotic surgery	Mean age/Pathology	Follow-up months	RLN injury definition postoperative time	MINORS score
Arora et al., 2016 [11]	Prospective/UK	RT: 16; BMI: 25.9 ± 5.4 kg m^2^; no. of females: 15; OT: 16; BMI: 27.7 ± 3.5 kg m^2^; no. of females: 14	TAA	RT: 42 ± 10.8 years; OT: 51.7 ± 15 years; follicular thyroid adenoma; follicular thyroid carcinoma; papillary thyroid carcinoma	4 ± 2 years (range, 6 months to 6 years)	Transient: Not defined; Permanent: Not defined	22
Cho et al., 2016 [12]	Retrospective/South Korea	RT: 109; BMI: 24.53 (16.63–37.77); no. of females: 94; OT: 109; BMI: 23.73 (17.58–33.37); no. of females: 91	BABA	RT: 41.78 ± 9.36; OT: 40.81 ± 10.84; papillary thyroid carcinoma	22–68	Transient: vocal cord palsy recovery in less than 6 months. Permanent: vocal cord palsy lasted more than 6 months.	18
Garstka et al., 2018a [13]	Retrospective/USA-France	RT: 56; BMI: 24.9 ± 4.9; no. of females: 54; OT: 46; BMI: 31.2 ± 13.5; no. of females: 40	TAA	RT: 38.6 ± 9.6; OT: 42.1 ± 16.4; Graves’ disease	6	Transient: vocal cord palsy recovery in less than 6 months. Permanent: vocal cord palsy lasted more than 6 months.	21
Garstka et al., 2018b [14]	Retrospective/USA	RT: 35; BMI: 25.0 ± 4.3; no. of females: 34; OT: 109; BMI: 32.4 ± 8.1; no. of females: 79	TAA	RT: 42.1 ± 12.5; OT: 52.7 ± 15.6; well-differentiated thyroid cancer	RT: 219.5 ± 183.9 days; OT: 296.7 ± 265.1 days	Transient: vocal cord palsy recovery in less than 6 months. Permanent: vocal cord palsy lasted more than 6 months.	22
Kang et al., 2012 [15]	Retrospective/South Korea	RT: 56; BMI: -; no. of females: 46; OT: 109; BMI: -; no. of females: 83	TAA	RT: 35.8 ± 9.1; OT: 46.1 ± 13.0; papillary thyroid carcinoma	12	Transient: Not defined; Permanent: Not defined	18
Kim et al., 2011 [16]	Retrospective/South Korea	RT: 69; BMI: 22.7 ± 2.7; no. of females: 63; OT: 138; BMI: 24.4 ± 3.1; no. of females: 104	BABA	RT: 41.3 ± 7.8; OT: 51.8 ± 8.9; papillary thyroid carcinoma	6	Transient: vocal cord palsy recovery in less than 6 months. Permanent: vocal cord palsy lasted more than 6 months.	18
Kim et al., 2016 [17]	Prospective /South Korea	RT: 112; BMI: -; no. of females: 106; OT: 117; BMI: -; no. of females: 106	BABA	RT: 38.9 ± 0.9; OT: 50.4 ± 1.0; papillary thyroid carcinoma	RT: 32.1 ± 6.3; OT: 32.8 ± 6.4	Transient: vocal cord palsy recovery in less than 6 months. Permanent: vocal cord palsy lasted more than 6 months.	22
Kwon et al., 2016 [18]	Retrospective/South Korea	RT: 44; BMI: 22.0 ± 2.2 (range 17.4–26.7); no. of females: 41; OT: 145; BMI: 23.5 ± 3.3 (range 16.8–37.9); no. of females: 123	BABA	RT: 35.1 ± 10.8; OT: 45.4 ± 14.8; Graves’ disease	RT: 33.8 ± 23.8; OT: 31.7 ± 22.7	Transient: vocal cord palsy recovery in less than 6 months. Permanent: persisted vocal cord palsy lasted at 6 months.	22
Landry et al., 2012 [19]	Retrospective/USA	RT: 25; BMI: 25; no. of females: 23; OT: 25; BMI: 26; no. of females: 21	TAA	RT: 50 (22–62); OT: 53 (24–75); papillary thyroid carcinoma	7	Transient: Not defined; Permanent: Not defined	22
Lee et al., 2012 [20]	Retrospective/South Korea	RT: 192; BMI: -; no. of females: 179; OT: 266; BMI: -; no. of females: 213	TAA	RT: 41.9 ± 9.2; OT: 48.7 ± 10.8; papillary thyroid microcarcinoma	29.1	Transient: Not defined; Permanent: Not defined	17
Noureldine et al., 2013a [21]	Retrospective/USA	RT: 12; BMI: 27.3 ± 6.8; no. of females: 10; OT: 13; BMI: 27.3 ± 5.5; no. of females: 10	TAA	RT: 40.3 ± 14.3; OT: 41.9 ± 15.2; Graves’ disease	5.5 months	Transient: vocal cord palsy recovery in less than 6 months. Permanent: persisted vocal cord palsy lasted at 6 months.	19
Noureldine et al., 2013b [22]	Retrospective/USA	RT: 24; BMI: 28.9; no. of females: 20; OT: 35; BMI: 34.1; no. of females: 21	TAA	RT: 45.4; OT: 52.6; follicular thyroid cancer; papillary thyroid cancer	12 ± 2.2 months	Transient: Not defined; Permanent: Not defined	22
Noureldine et al., 2013c [23]	Retrospective/USA	RT: 110; BMI: 29.4; no. of females:100; OT: 110; BMI: 30.9; no. of females: 81	TAA	RT: 46.4; OT: 51.9; thyroid nodule	Not defined	Transient: Not defined; Permanent: Not defined	22
Song et al., 2016 [24]	Retrospective/South Korea	RT: 25; BMI: 22.9 ± 2.9; no. of females: 24; OT: 41; BMI: 24.6 ± 3.1; no. of females: 30	GUA/GUAB	RT: 36.7 ± 10.5; OT: 47.5 ± 15.3; papillary thyroid carcinoma	RT: 29.0 ± 11.4; OT: 34.8 ± 14.5	Transient: vocal cord palsy recovery in less than 6 months. Permanent: vocal cord palsy lasted more than 6 months.	20
Tae et al., 2011 [25]	Retrospective/South Korea	RT: 41; BMI: -; no. of females: 39; OT: 163; BMI: -; no. of females: 118	GUA/GUAB	RT: 39.2 ± 10; OT: 51.7 ± 12.4; papillary thyroid carcinoma; nodular hyperplasia; follicular adenoma	3	Transient: Not defined; Permanent: Not defined	19
Tae et al., 2014 [26]	Retrospective/South Korea	RT: 62; BMI: 23.6 ± 3.7; no. of females: 61; OT: 183; BMI: 24.6 ± 3.8; no. of females: 141	GUA/GUAB	RT: 40.5 ± 9.6; OT: 51.4 ± 11.3; papillary thyroid carcinoma	27.9	Transient: Not defined; Permanent: Not defined	19
Tae et al., 2016 [27]	Retrospective/South Korea	RT: 185; BMI: 23.9 ± 5.0; no. of females: 166; OT: 185; BMI: 24.6 ± 4.5; no. of females: 165	GUA/GUAB	RT: 41.8 ± 9.1; OT: 42.6 ± 8.9; papillary thyroid carcinoma	RT: 42.3 ± 21.7; OT: 44.9 ± 19.2	Transient: vocal cord palsy recovery in less than 6 months. Permanent: vocal cord palsy lasted more than 6 months.	20
Woo et al., 2017 [28]	Prospective /South Korea	RT: 51; BMI: 22.8 ± 2.2; no. of females: 44; OT: 51; BMI: 23.7 ± 2.3; no. of females: 42	BAA	RT: 36.8 ± 11.5; OT: 42.4 ± 13.0; well-differentiated thyroid carcinoma or a benign thyroid nodule	10.4	Transient: vocal cord palsy recovery in less than 6 months. Permanent: vocal cord palsy lasted more than 6 months.	20

Meta-analysis

Transient RLN palsy

**Figure 2:**
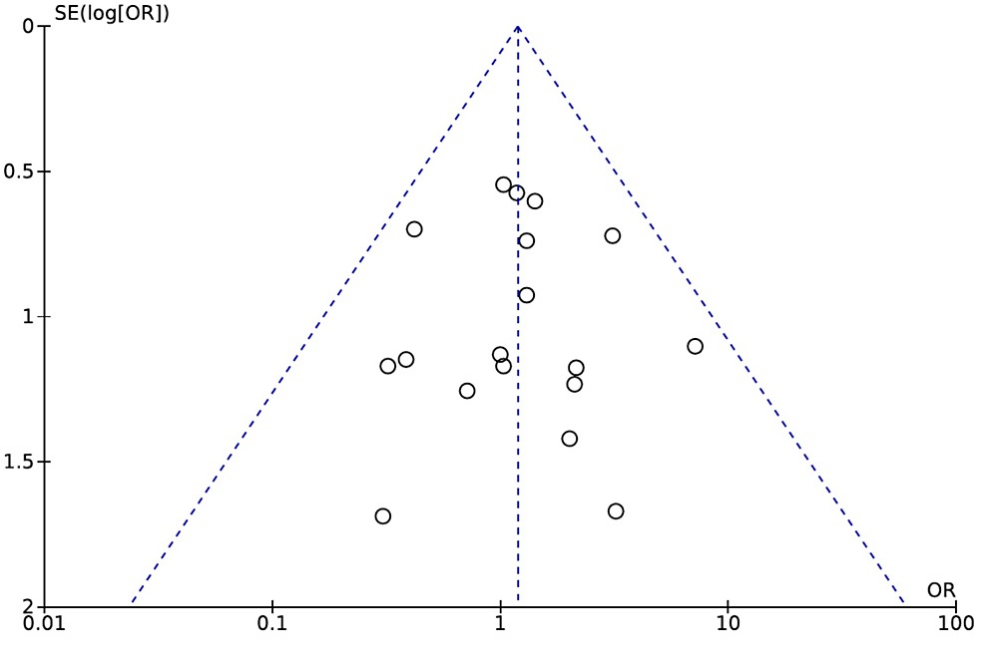
Funnel plot of the comparison of robotic versus open conventional thyroidectomy for transient RLN palsy RLN: Recurrent laryngeal nerve. Source: Authors of this study.

The funnel plot shows the comparison of robotic versus open conventional thyroidectomy for transient RLN palsy (Figure 2). The funnel plot of robotic versus OT for transient RLN palsy, which is shown in Figure 2, is symmetrical, indicating no publication bias.

**Figure 3:**
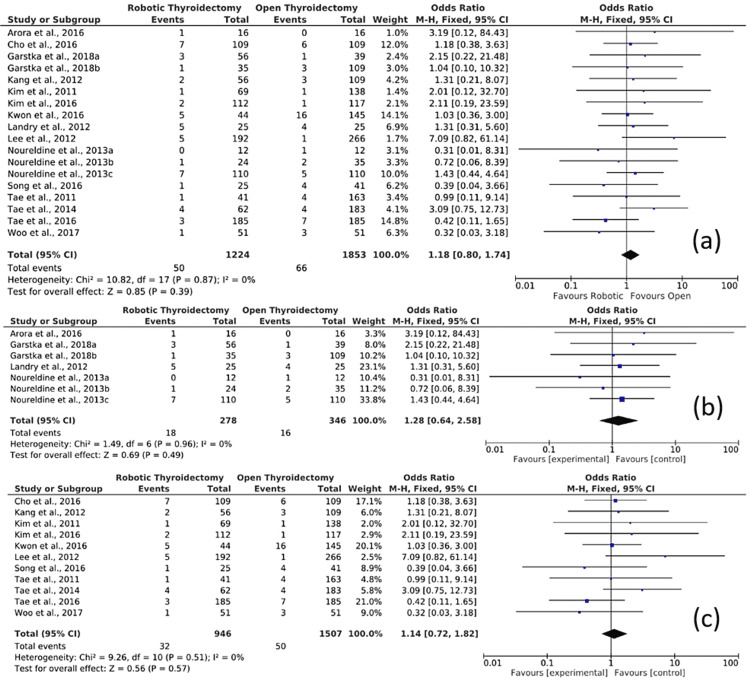
Forest plot of the comparison of robotic versus open conventional thyroidectomy for transient RLN palsy (a) Overall comparison of studies for transient RLN palsy, (b) subgroup analysis of studies from Western Countries for transient RLN palsy, and (c) subgroup analysis of studies from Asian Countries for transient RLN palsy. RLN: Recurrent laryngeal nerve. Source: Refs. [11-28].

Figure 3 displays the forest plot of the comparison of robotic versus open conventional thyroidectomy for transient RLN palsy, which shows the results of the meta-analysis. The meta-analysis of robotic versus OT for transient RLN palsy evaluated three outcomes.

First, the overall comparison of studies for transient RLN palsy is shown in Figure 3a. In this comparison, 18 studies were included. The results show 50 cases of transient RLN palsy from a total sample population of 1224 patients who had undergone RT and 66 cases of transient RLN palsy from a total sample population of 1853 patients who had undergone open conventional thyroidectomy. The Mantel-Haenszel method was used. The OR was 1.18, and the 95% confidence interval (95% CI) was 0.80-1.74. There is no statistically significant difference between RT compared to OT in the safety of RLN for transient palsy as the 95% CI was 1; hence, the results are not statistically significant. The heterogeneity I^2^ was 0%. The test for overall effect Z was 0.85 (P = 0.39).

Second, a subgroup analysis including only the studies conducted in the USA and Europe is shown in Figure 3b. In this comparison, seven studies were included. The results show 18 cases of transient RLN palsy from a total sample population of 278 patients who had undergone RT and 16 cases of transient RLN palsy from a total sample population of 346 patients who had undergone open conventional thyroidectomy. The Mantel-Haenszel method was used. The OR was 1.28, and the 95% CI was 0.64-2.58. There is no statistically significant difference between RT compared to OT in the safety of RLN for transient palsy as the 95% CI was 1; hence, the results are not statistically significant. The heterogeneity I^2^ was 0%. The test for overall effect Z was 0.69 (P = 0.49).

The third outcome is a subgroup analysis including only the studies conducted in Asia, which is shown in Figure 3c. In this comparison, 11 studies were included. The results show 32 cases of transient RLN palsy from a total sample population of 946 patients who had undergone RT and 50 cases of transient RLN palsy from a total sample population of 1507 patients who had undergone open conventional thyroidectomy. The Mantel-Haenszel method was used. The OR was 1.14, and the 95% CI was 0.72-1.82. There is no statistically significant difference between RT compared to OT in the safety of RLN for transient palsy as the 95% CI was 1; hence, the results are not statistically significant. The heterogeneity I^2^ was 0%. The test for overall effect Z was 0.56 (P = 0.57).

Permanent RLN Palsy

**Figure 4:**
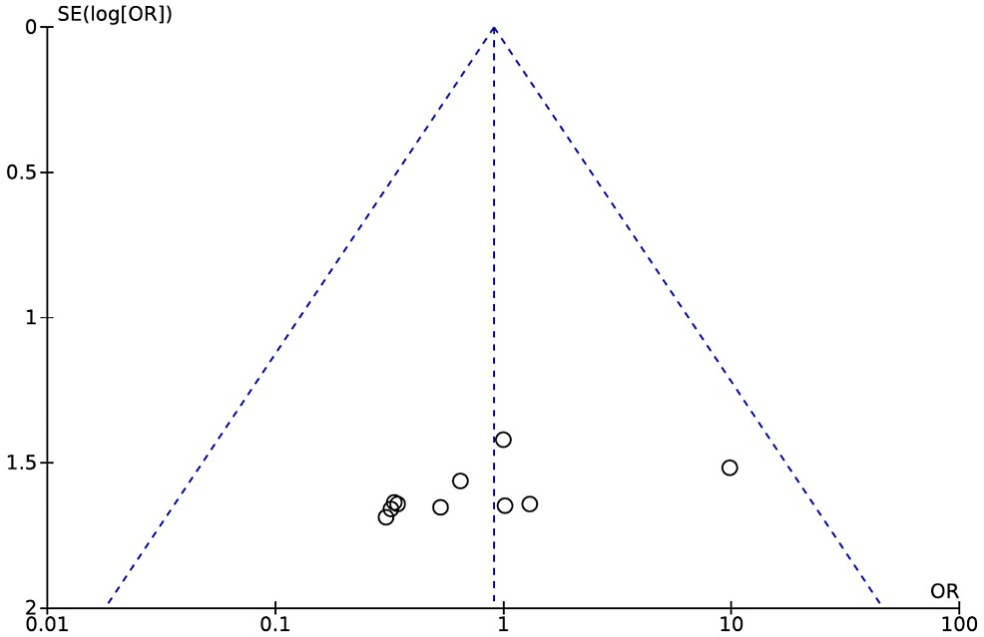
Funnel plot of the comparison of robotic versus open conventional thyroidectomy for permanent RLN palsy RLN: Recurrent laryngeal nerve. Source: Authors of this study.

The funnel plot of robotic versus OT for permanent RLN palsy, which is shown in Figure 4, is symmetrical, indicating no publication bias.

**Figure 5:**
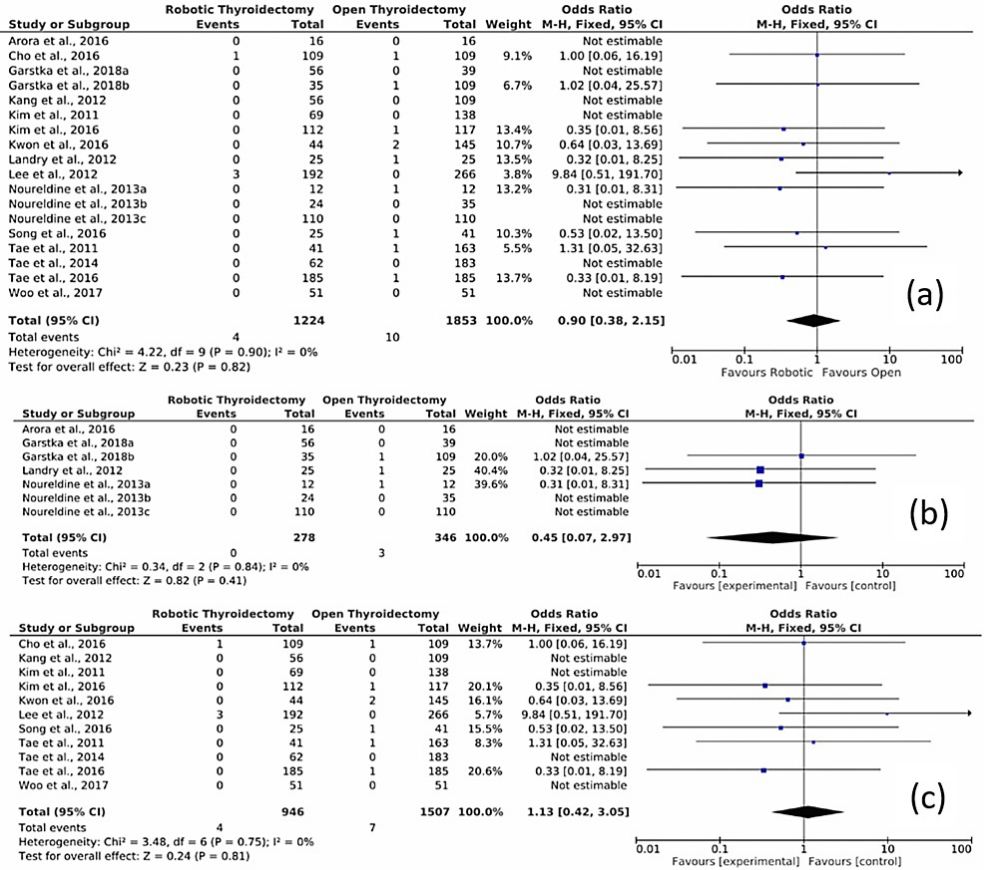
Forest plot of the comparison of robotic versus open conventional thyroidectomy for permanent RLN palsy (a) Overall comparison of studies for permanent RLN palsy, (b) subgroup analysis of studies from Western Countries for permanent RLN palsy, and (c) subgroup analysis of studies from Asian Countries for permanent RLN palsy. RLN: Recurrent laryngeal nerve. Source: Refs. [11-28].

Figure 5 shows the results of the meta-analysis. In the meta-analysis for robotic versus OT for permanent RLN palsy, three outcomes were evaluated.

First, the overall comparison of studies for permanent RLN palsy, which is shown in Figure 5a, was assessed. In this comparison, 18 studies were included. The results show four cases of permanent RLN palsy from a total sample population of 1224 patients who had undergone RT and 10 cases of permanent RLN palsy from a total sample population of 1853 patients who had undergone open conventional thyroidectomy. The Mantel-Haenszel method was used. The OR was 0.90, and the 95% CI was 0.38-2.15. There is no statistically significant difference between RT compared to OT in the safety of RLN for permanent palsy as the 95% CI was 1; hence, the results are not statistically significant. The heterogeneity I^2^ was 0%. The test for overall effect Z was 0.23 (P = 0.82).

Second, a subgroup analysis including only the studies conducted in the USA and Europe, is shown in Figure 5b. In this comparison, seven studies were included. The results show zero cases of permanent RLN palsy from a total sample population of 278 patients who had undergone RT and three cases of permanent RLN palsy from a total sample population of 346 patients who had undergone open conventional thyroidectomy. The Mantel-Haenszel method was used. The OR was 0.45, and the 95% CI was 0.07-2.97. There is no statistically significant difference between RT compared to OT in the safety of RLN for transient palsy as the 95% CI was 1; hence, the results are not statistically significant. The heterogeneity I^2^ was 0%. The test for overall effect Z was 0.82 (P = 0.41).

The third outcome is a subgroup analysis including only the studies conducted in Asia, which is shown in Figure 5c. In this comparison, 11 studies were included. The results show four cases of permanent RLN palsy from a total sample population of 946 patients who had undergone RT and seven cases of permanent RLN palsy from a total sample population of 1507 patients who had undergone open conventional thyroidectomy. The Mantel-Haenszel method was used. The OR was 1.13, and the 95% CI was 0.42-3.05. There is no statistically significant difference between RT compared to OT in the safety of RLN for transient palsy as the 95% CI was 1; hence, the results are not statistically significant. The heterogeneity I^2^ was 0%. The test for overall effect Z was 0.24 (P = 0.8).

Discussion

We included 18 non-randomized studies in this meta-analysis, and we performed research in electronic databases such as PubMed, CINAHL, Cochrane CENTRAL, MEDLINE, and ScienceDirect in September 2022. Further research was performed during January 2024 in the Scopus database. We conducted a systematic review and meta-analysis following the PRISMA guidelines. We assessed the risk of bias and extracted the data needed for each study included. In this meta-analysis, we performed the following comparisons: first, the overall analysis of robotic versus open conventional thyroidectomy for transient RLN palsy and robotic versus open conventional thyroidectomy for permanent recurrent laryngeal nerve RLN palsy. Second, we performed subgroup analysis for transient and permanent RLN palsy between the USA/Europe and Asian studies for each outcome.

In the overall comparisons, we included 18 studies for each outcome. Comparable effects were observed without statistical significance. A subgroup analysis was performed for each outcome separately. Studies conducted in the USA/Europe were separately analyzed for transient and permanent palsy. In this subgroup, seven studies were included. Likewise, no statistical significance was observed. Subgroup analysis was performed for transient and permanent RLN palsy separately including the studies conducted in Asia. In this analysis, 11 studies were included. Again, we did not observe statistical significance.

We observed a variety of RT approaches. Four studies [12,16-18] used the bilateral axillo-breast approach (BABA), one study [28] used the bilateral axillary approach (BAA), nine studies [11,13-15,19-23] used the transaxillary approach (TAA), and another four studies [24-27] used the gasless unilateral axillary or an axillo-breast approach (GUA/GUAB). No major flaws were observed in the quality assessment of the included studies according to the MINORS scale [7]. No statistically significant results were observed in the two comparisons. We did not observe a statistically significant difference in the safety of RLN comparing robotic and conventional OT.

The method of BABA RT is like an endoscopic technique at the point of insertion. The 30-degree endoscope is placed in the circumareolar area on the side of the lesion through a 12 mm port [16]. The Harmonic scalpel is placed on the circumareolar area on the contralateral side through an 8 mm port [16].

According to Noureldine et al., [22] for the procedure of TAA, a subcutaneous flap is raised in a subplatysmal plane. A retractor is placed under the sternal head of the sternocleidomastoid and strap muscles. A dissection is performed like a conventional technique.

Woo et al.'s study [28] suggested a novel approach, in which the authors [28] suggested a BAA, a technique that does not require breast incision. The procedure of the BAA is similar to that of the BABA. The patient is positioned supine with the neck extended under general anesthesia. Axillary trocars of 8-12 mm are inserted bilaterally. The robot is fixed, the Harmonic scalpel is inserted through the contralateral inner port, and the camera is inserted through the ipsilateral inner port.

For GUA/GUAB, the thyroidectomy proceeds with flap elevation and robot system docking [25]. For the axillo-breast approach, a second skin incision on the circumareolar margin of the breast is performed. Three arms are inserted through the axillary incision port, such as the dual-channel 30-degree endoscope, the Harmonic curved shears, and Maryland forceps [25].

In general, postoperative vocal cord palsy is defined as the presence of an immobile vocal cord or the decrease in the movement of the affected vocal cord during phonation [29]. In most of the studies included in the review, transient RLN palsy was defined as vocal cord palsy recovered in less than six months. Permanent RLN palsy was defined as persistent vocal cord palsy that lasted up to or more than six months.

Notwithstanding that, other meta-analyses compared robotic and conventional thyroidectomy in a variety of outcomes, but we preferred to investigate in depth only the RLN as a postoperative complication because we believe that RLN affects the quality of the patient’s life and warrants significant attention.

Although our results are comparable to previous meta-analyses, we observed a significant lack of data from Western countries and specifically from Europe. We describe relevant meta-analyses to compare our results and to show the need for more studies to be conducted from Western health centers.

According to Liu et al., [30], no statistically significant difference was observed for transient RLN palsy between RT and OT groups (RR 1.02, 95% CI 0.80-1.30, P = 0.88, I^2^ = 0%), and for permanent RLN palsy, the analysis showed that RT group had a lower incidence compared to OT group although the results were not statistically significant (RR 0.57, 95% CI 0.29-1.12, P = 0.10, I^2^ = 0%). Furthermore, the study [30] concluded that RT is a safe approach with significant superiority in reducing intraoperative damage and improving patients’ quality of life compared to OT. The review included 59 studies, of which 10 were performed in the USA, one in the United Kingdom, and the rest in the Asian continent.

Pan et al., [31] concluded that RT is as safe as OT, and the adverse events and complications of RT were comparable with OT. As far as transient RLN palsy OR was 1.11, 95% CI 0.74-1.66, P = 0.63, and permanent RLN palsy OR was 1.05, 95% CI 0.45-2.43, P = 0.92, the results were not statistically significant. Consequently, there was no significant difference between RT and OT. From a total of 23 included studies, 22 were conducted in South Korea and one in the USA. In the comparison performed for transient RLN palsy, 16 studies were performed in South Korea and one in the USA.

In a meta-analysis by Son et al., [32], transient RLN palsy was OR 1.39, 95% CI 0.81-2.37, P = 0.23, and permanent RLN palsy was OR 3.43, 95% CI 0.82-14.42, P = 0.09. The authors concluded there were no significant differences between RT and OT groups. Of the 14 studies included, only two were conducted in the USA.

Shan and Liu [33] performed a meta-analysis comparing the BABA to RT and conventional thyroidectomy. They included 11 studies, of which one was conducted in China and the other was in Korea. As for transient RLN palsy, OR was 1.22, 95% CI = 0.76-1.96, P = 0.40, and for permanent RLN palsy, OR = 0.53, 95% CI = 0.14-2.03, P = 0.36.

In a newer meta-analysis of Zhou et al., [34] published in 2023, a comparison of the transoral thyroidectomy vestibular approach versus conventional OT was performed. Despite the specific research question, only one study was conducted in the USA and one in Mexico. The rest of the studies were conducted in China, Korea, and Thailand.

In a prospective study by Arora et al., [11] conducted in the UK, the authors concluded that there is a substantial difference between Western patients and South East Asian patient population, although RT is achievable and safe in UK patients.

Furthermore, the authors stated that the majority of publications on RT are conducted in South Korea and the Far East, and they provide some reasons for this observation. First, in the Far East, a cosmetic problem such as a horizontal neck scar is negatively perceived, so RT, which leaves no scar, is preferred. Another explanation is the increased incidence of thyroid cancer in South Korea compared to the UK and USA and the developed thyroid cancer screening program of the country. Furthermore, papillary microadenomas identified are suitable for RT because the thyroid gland remains in normal size. Moreover, according to the study [11], another reason is the financial compensation for the procedure by the healthcare systems. Thyroidectomy, in USA and UK, is financially supported by the extent of the operation performed. By contrast, in Korea, trans-axillary RT is compensated four times more than the open conventional thyroidectomy and twice the endoscopic thyroidectomy. Additionally, the anatomical characteristics are different between populations. South Korean patients are slimmer, with lower body mass index, a factor that makes them suitable for transaxillary RT in contrast to the Western population patients, who have higher body mass index, and as a result, the robotic procedure is more difficult.

According to Kim et al., [17], thyroid cancer is more often in young women, and hypertrophic scarring after thyroid surgery is more probable to occur in Asians. Even though endoscopic thyroid surgery has a very good cosmetic result in comparison to OT, the endoscopic procedure has limitations due to two-dimensional view and non-flexible instruments.

Limitations of the Evidence Included and the Review Process

In this review, although we followed specific criteria, there are some limitations. First, we focused only on postoperative complications of the RLN. Second, despite the comprehensive research in electronic databases, we may miss some relevant studies. Third, we only analyzed the postoperative complications of RLN, not considering the existing pathology of the thyroid gland and the approach of RT. Fourth, most of the studies were conducted in South Korea. This means that there are differences in patient characteristics from other regions of the world. Fifth, we observed heterogenicity among included studies, such as in follow-up time and RT approach.

In this review, an existing factor of possible bias was faced because most of the studies were performed in South Korea. In general, as we observed in other relevant meta-analyses, studies performed in South Korea outnumber the studies performed in other regions of the world. This phenomenon may create a bias because patients in East Asia may have different characteristics from patients in Europe and America.

Therefore, the results should be interpreted with caution as we do not have worldwide data to integrate. Considering the data and results included in this review as well as from other meta-analyses, we observed a limitation of comparative studies in the Western population in the literature.

Implications of the Results for Practice, Policy, and Future Research

In summary, future research could concentrate on larger cohort studies and comparative studies to reduce the risk of bias. Furthermore, longer follow-up times between studies and examinations of voice recovery are advisable. In addition, quality of life (QoL) and patient satisfaction should be priorities of future research. Moreover, it is important to consider the patient’s views on the treatment. The feedback from patients contributes to the improvement of the treatment [35].

Lastly, it is important to note the lack of studies performed on the Western population, especially in Europe. Although many studies were performed in the USA, most of the data published was from East Asia, specifically from South Korea. More comparative studies are needed on the Western population, specifically Europe, as we observed a lack of data in this region. Given this limitation, we expect more comparative studies to be performed from other regions of the world.

## Conclusions

In this meta-analysis, first, the overall comparison of RT and OT for transient RLN palsy as a postoperative complication showed that both techniques are comparable, although the results were not statistically significant. Furthermore, the overall comparison of RT and OT for permanent RLN palsy as a postoperative complication showed that both techniques are comparable, although the results again were not statistically significant.

Second, the subgroup comparisons for transient and permanent RLN palsy between the USA/Europe and Asian countries showed no statistically significant results. Considering there is not a statistically significant difference between the two approaches for RLN safety and due to the limited number of studies from Western countries, the results should be considered with caution. Both OT and RT have advantages and disadvantages; hence, important factors such as the patient’s body characteristics and the existing thyroid pathology should be kept in mind. Furthermore, the surgical approach in every case should be considered; therefore, patient selection for RT or OT is a principal factor for best results. More comparable studies are needed on the Western population.
